# The Impact of Exercise Intensity Feedback Using Technology for Children During Active Play: Pilot Study

**DOI:** 10.2196/11327

**Published:** 2018-11-23

**Authors:** Madison Blake, Martin Sénéchal, Megan Comeau, Spencer Smith, Danielle Bouchard

**Affiliations:** 1 Cardio-metabolic Exercise & Lifestyle Laboratory Faculty of Kinesiology University of New Brunswick Fredericton, NB Canada

**Keywords:** biofeedback, exercise intensity, physical activity

## Abstract

**Background:**

Most children do not engage in enough exercise at the recommended intensity. Using technological devices may increase the time children spend at greater intensities while exercising.

**Objective:**

This study aimed to determine if children who are receiving instant feedback on their exercise intensity using technology would spend more time in moderate-vigorous intensity (≥70% of maximum capacity) during active play sessions. It also aimed to explore if the children’s physical characteristics were associated with the average percentage of maximal heart rate (HR) reached during sessions.

**Methods:**

Participants were asked to wear a HR monitor, attached around their chest, for 4 sessions out of the 15 sessions offered. Twenty children aged 5 to 11 years received feedback for 2 random sessions. When receiving feedback, color-coded intensity based on HR was projected onto a wall. Green corresponded to moderate intensity (≥70% of max HR) and red corresponded to a HR below moderate intensity. Age, anthropometric measures, muscle strength, body composition, physical activity level, and fitness level were measured.

**Results:**

The average percentage of maximal HR during a session was similar whether feedback was provided (70.7%, SD 6.4%) or not (71.1%, SD 4.1%) with *P*=.93. No personal characteristics were associated with the average intensity recorded during the exercise sessions.

**Conclusions:**

Receiving instant exercise intensity feedback is not associated with a higher proportion of time spent at moderate intensity or above in children aged 5 to 11 years when involved in an active play program. Personal characteristics are not associated with the intensity recorded when participating in an active play program.

## Introduction

Moderate intensity exercise is often defined based on a percentage of estimated maximum heart rate (HR), estimated by subtracting a person’s age in years from 220, ranging from 50% to 70% [1]. Polar Canada [2] characterizes moderate-vigorous intensity as 70% or higher of an individual’s maximum HR. Physical activity performed at moderate-to-vigorous intensity is associated with a lower risk of (1) obesity, (2) elevated cholesterol levels, (3) hypertension, and (4) metabolic syndrome in children [3]. A small proportion of Canadian children meet the World Health Organization physical activity guidelines which recommend 60 minutes per day of moderate-vigorous intensity physical activity [4]. Children involved in organized activities spending only 30% of the time in moderate intensity or more during a typical extracurricular session are often not reaching the physical activity guidelines throughout the week [5].

The ability to monitor one’s intensity via technology could potentially motivate children to stay within the most beneficial intensities [6]. A whole body of evidence suggests that technologies provide stimulation for children and in turn, they are spending immense amounts of time using technology [7,8]. Children aged 8-10 years spend approximately eight hours per day using technology [9]. Health tracking devices have become more prevalent in society, even for children [10]. These devices provide feedback when performing physical activities and may increase a participant’s motivation [11]. For example, studies have shown when adolescents have constant access to physical activity trackers, their running distance, energy expenditure, and time spent in moderate to vigorous intensity increases [10,11]. However, to the best of our knowledge, only one study has used a similar technology of instant feedbacks to increase time spent at moderate intensity or higher [12]. This study was conducted in Australia and had children aged 11-13 years wear a HR monitor during their physical education classes. One group of children had constant access to feedback for 5 weeks via a Polar HR watch, and the control group did not. After each class, the children were asked to estimate how many minutes they spent in moderate to vigorous intensity and both groups were unable to estimate the number of minutes adequately. This shows that intensity perception is a difficult concept for children to understand [1,10,12]. While several studies have looked at the effect of wearable biofeedback devices with adolescents, to the best of our knowledge there have been no studies looking at the effects of biofeedback on intensity in young children. This may be because the literature suggests children do not develop cognitively and struggle to understand incoming stimuli at a young age [12]. However, even adolescents struggle to identify intensity using wearable devices, and there is a wide array of devices being targeted towards youth as they spend large quantities of time using technology [9,12].

The main objective of this study was to determine if children, as young as 5 years of age, would perform at moderate intensity or above for a longer period when receiving feedback of their intensity via a color-coded projection on the wall compared to children not having feedback in the same session. We also aimed to explore if children’s physical characteristics were associated with the average percentage of maximal HR reached during sessions.

## Methods

### Overview

Children between the ages of 5 and 11 were recruited through a local active play program lasting 12 weeks in which children engaged in 60 minutes of physical activity per session for 2 evening per week. Active play is defined as a form of gross motor or total body movement in which young children exert energy in a freely chosen, fun, and unstructured manner [13]. The current program was designed to engage children in the simple pleasures and benefits of regular physical activity, with a variety of fun interactive and noncompetitive physical activities intended to foster self-esteem, confidence, a positive self-image and the joy of being physically active. The program included games such as tag, “red light green light” and “fishes and whales.” These games include sprinting, switching direction, and starting and stopping. The program was semistructured so the coaches had games planned but free play with ropes, balls and other gym equipment was always an option for the children, as well as suggesting alternative activities.

### The Intervention

Before every session, the research assistant would open an envelope identifying which participants were selected to wear a HR monitor. This was selected randomly using the select case function in the statistical software SPSS (International Business Machines Corporation, New York, United States). If a participant missed a session, the randomization was postponed for the next session.

Participants were asked to wear a HR monitor, attached around their chest, for 4 sessions out of the 15 sessions offered. However, they only received feedback for 2 of the 4 sessions during which they wore a HR monitor. For the 2 sessions where participants received feedback, their HR was displayed on a wall in the gym. This method was used rather than a wearable monitor to create a group experience and encourage all the children to be “green” indicating they had all reached moderate intensity. The wall made it easy for the children to monitor their heart rate and for the researchers to explain the concept to the children. Each child was associated with a number to ensure anonymity, and their goal was to maintain their HR at moderate intensity (70% of maximum HR [14]) as indicated by displaying the number in green. Displaying the number in red indicated that their HR was below moderate intensity. For the other 2 sessions, the participants wore the equipment but did not receive feedback. The participant’s response to feedback was analyzed on a per session basis to account for the variability in the session’s activities. Parents signed a consent form and children signed an assent form before the study began. A research ethics board approved this project.

### Measuring the Children’s Characteristics

Children’s anthropometrics, handgrip strength, body composition, cardiorespiratory fitness, and physical activity levels were measured to assess if these characteristics were associated with the average percent of maximal HR the children reached during the 4 sessions.

Anthropometrics measures and grip strength were collected to describe the sample of participants according to the Canadian Society of Exercise Physiology Protocol. These were obtained during 1 of the sessions when the participants were not wearing any equipment. Height was measured to the nearest 0.5 cm using a SECA stadiometer (SECA, Hamburg, Germany) and body weight was measured to the nearest 0.1 kg on a SECA model number 213 calibrated column scale. Waist circumference was taken with an anthropometric tape and measured to the nearest 0.5 cm at the upper lateral border of the iliac crest after the participant had crossed their arms over their chest [15]. Grip strength was measured with a hand dynamometer as the participant held the grip between their fingers and the base of their thumb. Two trials were performed on each hand, and the highest score of all trials was entered and analyzed.

Participants’ body compositions were estimated using the Bod Pod (Cosmed, Concord, California, United States.). The Bod Pod was calibrated following standard protocol [16]. The participants wore minimal clothing and a bathing cap while sitting still in the Bod Pod. The thoracic gas volume was estimated, and the Brozek equation [17] was used to estimate the fat mass and muscle mass.

Cardiorespiratory fitness was estimated using the 20-meter shuttle test [18]. As per protocol, children ran between 2 lines 20 meters apart reaching the line before the sound of a beep. Every minute the participants were required to increase their speed by 0.5 km/h [18]. The children were first given a warning when they did not reach the line on time and eliminated after the second consecutive time. Cardiorespiratory fitness was estimated using a published equation developed for this population [19].

Finally, participants were asked to wear an SC-StepRx pedometer (StepsCount Inc, Deep River, Canada) for 7 days to capture steps per day and time spent in moderate-vigorous intensity. Intensity was estimated based on cadence thresholds: 110 for moderate and 130 for vigorous [20]. Children were given the pedometers on a Tuesday or Thursday after the exercise session and were asked to give it back after the session on the following week. Therefore, the information included sessions in the program. A minimum of 4 days of wear time was required to be included in the analysis. Averages were used to control for varying durations in wear time.

### Statistics

Due to the small sample size, nonparametric tests were used. Nonparametric Mann-Whitney *U* tests were performed between the children’s average percentage of maximal HR while having feedback and the children’s average percentage of maximal HR while not receiving feedback for each session. The Spearman rank correlation between the individual’s average percentage of maximal HR during all 4 sessions and participant’s characteristics was computed.

## Results

### The Projected System

**Figure 1:**
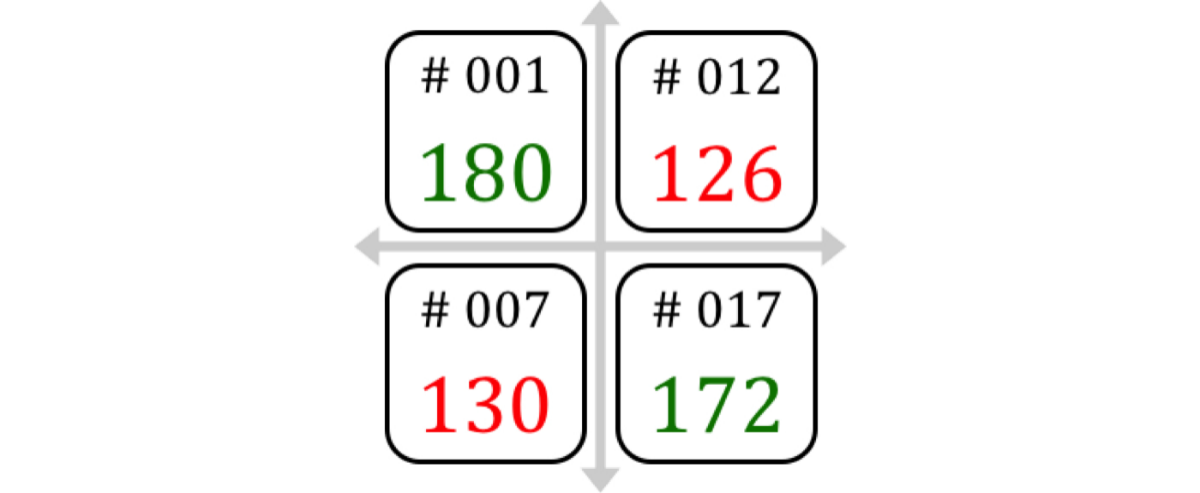
The projected system used to display biofeedback to the children.

Feedback was provided by showing a display on a wall (see Figure 1). The children knew their identification number, and their heart rate was color-coded to indicate their intensity. They understood that green was the optimal zone and red meant they were below optimal intensity and if they worked harder they would reach the green zone.

### The Children’s Characteristics

The median age was 7 years, with slightly fewer boys (9/20, 45%) than girls (11/20, 55%) participating in the study (see Table 1). The median daily steps per day were 12,051 steps per day and 276.6 minutes in that week was spent in moderate to vigorous intensity.

No significant relationships were found (*P*>.05) between the participants’ average percent of maximal HR reached during the active play sessions and the measured participants’ characteristics (see Table 2).

### The Intervention

**Figure 2:**
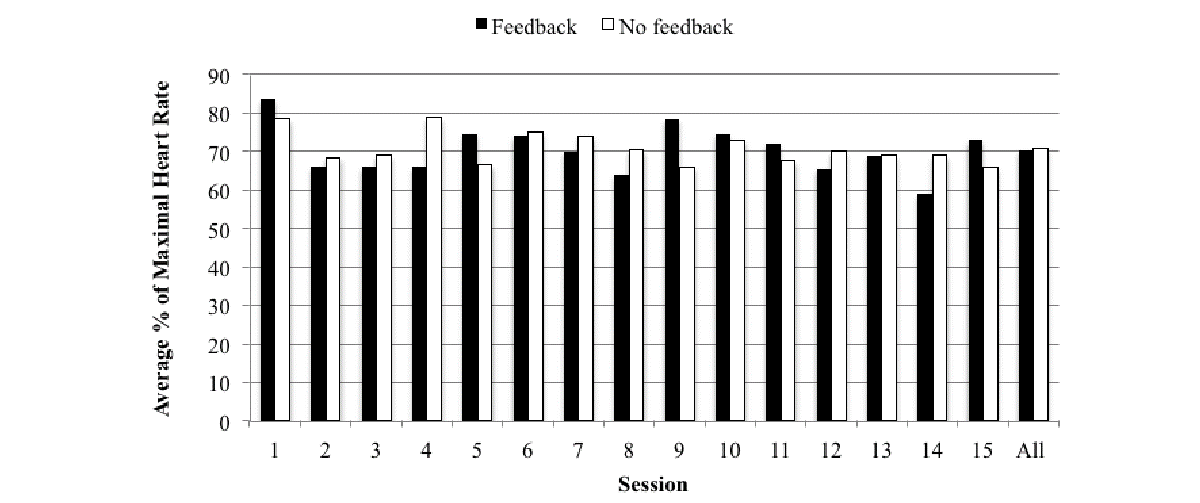
The association between the median percentage of maximal heart rate with and without feedback.

No significant difference (*P*=.93) between the average percentage of maximal HR of the participants who received feedback and the average percentage of maximal HR of participants who did not receive feedback during the recorded sessions, with an effect size of –0.004 standard deviation units (see Figure 2). The x-axis represents the session in which data was recorded, and the children’s average percent of maximal HR based on their estimated maximal HR is plotted on the y-axis. Nonparametric independent *t* tests were used to determine that there was no significant difference (*P*>.05) between the feedback and nonfeedback group for all 15 sessions. The groups were compared on a session by session basis to control for the variability of each session. This ensures the feedback and nonfeedback group were engaging in the same activities. The median percentage of HR max during all sessions was 71%, and 13/20 (65%) children had an average of 70% of their HR max for all sessions.

**Table 1:** A summary of the children’s characteristics (N=20).

Characteristics		Value
Age (years), median (IQR^a^)		7.0 (6.0-8.0)
**Gender, n (%)**		
	Male	9 (45)
	Female	11 (55)
Estimated VO_2max_ (mL/kg/min), median (IQR)		44.4 (43.5-48.0)
Height (cm), median (IQR)		130.0 (127.0-133.5)
Weight (kg), median (IQR)		25.9 (24.5-33.8)
Waist circumference (cm), median (IQR)		61.0 (56.0-67.5)
Grip strength (kg), median (IQR)		11.0 (8.0-13.0)
Fat mass (%), median (IQR)		21.6 (16.7-27.7)
Average daily steps^b^, median (IQR)		12,051 (9,649-15,106)
Weekly MVPA^c^ (min)^b^, median (IQR)		276.6 (197.4-356.1)

^a^IQR: interquartile range.

^b^N=9.

^c^MVPA: moderate-to-vigorous physical activity.

**Table 2:** The relationship between the children’s percent of maximum heart rate and their characteristics using the Spearman rank correlation (ρ).

Characteristic	ρ	*P* value
Age (years)	.27	.26
Weight (kg)	.19	.44
Height (cm)	.30	.21
Waist circumference (cm)	.058	.82
Grip strength (kg)	.055	.82
Fat mass (%)	–.022	.93
Physical activity level	–.18	.58
Fitness level	–.096	.69

**Figure 3:**
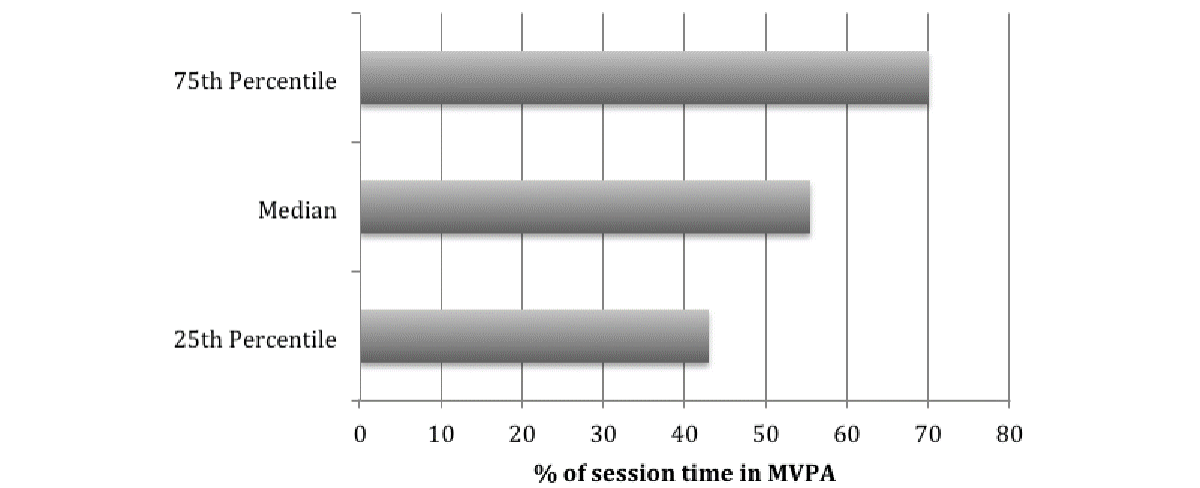
The proportion of time spent at moderate intensity or greater for all sessions. MPVA: moderate-to-vigorous physical activity.

Figure 3 displays the median proportion of time spent in moderate-vigorous intensity during the recorded sessions. This includes all 15 sessions with data collected from 20 children each wearing a heart rate monitor 4 times, twice with feedback and twice without feedback. Therefore, illustrating the time children spent engaging in moderate-to-vigorous physical activity (MVPA) during the active play program regardless of biofeedback. The median proportion of time spent in MVPA during all active play sessions was 20.5 minutes, representing an average of 55.5% per session.

## Discussion

### Principal Findings

The main objective of this study was to determine if children, as young as 5 years of age would spend more time at moderate to vigorous intensity when receiving feedback of their intensity via a color-coded HR projected onto a wall compared to children not receiving feedback in the same session. Although 55.5% of the time was spent at moderate intensity or above, the present findings indicate that when young children are receiving instant feedback during active play, they were not spending more time in MVPA compared to those who are not receiving feedback. We also aimed to explore if children’s physical characteristics were associated with the average percentage of maximal HR reached during the sessions. The participants’ characteristics were not significantly associated with the average percentage of maximal HR they reached during the 4 monitored active play sessions.

The ability to identify exercise intensity is a complex task for anyone including children [12,21,22]. The median age of the participants involved in this study was 7 years. Their age may have contributed to their inability to perceive the intensity as previous research has suggested that children do not develop the ability to logically interpret surrounding stimuli, until 11 years of age [12,23]. Therefore, it is possible that children involved in this study did not have the cognitive ability to interpret and respond to feedback related to the exercise intensity, despite the extensive time young children now spend using technology. A study by Gaudet et al [10] reported that the Prochaska’s stages of change were influencing the ability to be receptive to feedback and do more activity at moderate intensity with adolescents when in the stage of action. The participants’ stage of change in the current study is unknown and should have been asked of the children and their parents. The children in this study were also quite active based on their daily step count, averaging more than 12,000 steps per day. It is possible that different results would have been observed in a curriculum instead of during an after-school program that tends to attract active kids [5]. Even if the feedback was not associated with greater intensity, it is important to note that 55.5% of the sessions were spent at the moderate-vigorous intensity. This is important because most traditional physical education classes report [24] that the average is only 39.4% for boys and 29.1% for girls in physical education classes. Additional studies [25] have shown that young children enjoy physical activity more when the activity is noncompetitive, and there is a choice involved compared to structured activities. Potentially, enjoyment contributed to the children reaching an average of 70% of their maximal HR regardless of whether they were receiving feedback.

Of the characteristics that were measured, none were associated with the average percentage of maximal HR reached during sessions, regardless of feedback or not. Regarding body weight, it seems that intensity was not influencing the reached intensity during sessions when performing the active play. This result is similar to a study conducted with children aged between 6 and 9 years that found that nonobese and children living with obesity had similar heart rates during active play sessions [1]. Prior research has shown that children living with obesity perceive themselves to be less competent than their peers, and would feel more comfortable engaging in active play than in a structured athletic setting [1].

Children are often more active during physical education classes if they have a high ability level and low body mass index [24]. Since the participants’ physical characteristics did not affect the children’s average intensity, perhaps active play should be encouraged in different settings to increase fitness level.

### Limitations

There were some limitations to this study. First, this study used a small sample size. The children were also young, considered active and were voluntarily attending the program. While the activity sessions were an hour in length, the children’s heart rates were only recorded for an average of 37 (SD 4) minutes. Second, the sample was not randomized, and a crossover design was not used. Each session was different. This means that the children did not necessarily engage in the same activities when they had or did not have feedback. The children receiving feedback were not isolated from the children not receiving feedback. Therefore, the children not receiving feedback may have been mirroring the intensity of the children who were receiving feedback. However, physical activity was objectively measured using blinded pedometers. The color-coded projection on the wall was novel for the children and the active play program allowed for a well-supervised physical activity intervention.

### Conclusions

Providing instant feedback about their intensity for children with an average age of 7 years does not significantly increase their intensity when engaging in an active play session. However, it is important to note that during the active play sessions, children spent 55.5% of the time at moderate to vigorous intensity. Further research should examine the effects of providing feedback on exercise intensity when performing active play with older children with a broader range of physical activity levels.

## Abbreviations

HR: heart rate
IQR: interquartile range
MVPA: moderate-to-vigorous physical activity
